# The Role of Imaging in Aortic Valve Disease

**DOI:** 10.1007/s12410-016-9383-z

**Published:** 2016-06-07

**Authors:** Russell J. Everett, David E. Newby, Andrew Jabbour, Zahi A. Fayad, Marc R. Dweck

**Affiliations:** BHF/Centre for Cardiovascular Science, University of Edinburgh, Chancellor’s Building, 49 Little France Crescent, EH16 4SB Edinburgh, UK; St Vincent’s Hospital, Sydney, Australia; Icahn School of Medicine at Mount Sinai, New York, USA

**Keywords:** Valve, Stenosis, Regurgitation, Magnetic resonance imaging, Echocardiography, Computed tomography, Positron emission tomography

## Abstract

**Purpose of Review:**

Aortic valve disease is the most common form of heart valve disease in developed countries. Imaging remains central to the diagnosis and risk stratification of patients with both aortic stenosis and regurgitation and has traditionally been performed with echocardiography. Indeed, echocardiography remains the cornerstone of aortic valve imaging as it is cheap, widely available and provides critical information concerning valve hemodynamics and ventricular function.

**Recent Findings:**

Whilst diagnostic in the vast majority of patients, echocardiography has certain limitations including operator variability, potential for measurement errors and internal inconsistencies in severity grading. In particular, low-gradient severe aortic stenosis is common and challenging to diagnose. Aortic valve imaging may therefore be improved with alternative and complimentary multimodality approaches.

**Summary:**

This review investigates established and novel techniques for imaging both the aortic valve and the myocardial remodelling response including echocardiography, computed tomography, cardiovascular magnetic resonance and positron emission tomography. Moreover, we examine how the complementary information provided by each modality may be used in both future clinical practice and the research arena.

## Introduction

Aortic valve disease is the most common valvular heart disease in the developed world [1]. In particular, calcific aortic stenosis is responsible for considerable morbidity and mortality [2]. *Aortic stenosis* (AS) was once thought to be related to simple “wear and tear” as a result of advancing age but is increasingly understood to be a highly regulated process with some similarities to atherosclerosis. An initiating event is believed to cause endothelial damage, inflammatory cell infiltration and initiation of calcification. A progressive cycle of calcium deposition in the valve leaflets then occurs leading to an inexorable march towards severe aortic stenosis and the development of symptoms and heart failure unless aortic valve replacement (AVR) is performed [3]. *Aortic regurgitation* (AR) is common in calcific aortic valve disease but may also be caused by other pathology affecting the valve, such as endocarditis, or the aortic root, causing functional regurgitation as in hypertension, Marfan syndrome, annulo-aortic ectasia, collagen vascular disease and aortic dissection.

In both aortic stenosis and regurgitation, imaging of the aortic valve is critical in establishing a diagnosis, grading severity and informing the timing of valvular intervention. In addition, the importance of the myocardial remodelling response to these forms of valve disease is increasingly appreciated [4]. Aortic stenosis leads to a pressure-overloaded left ventricle, resulting in the left ventricular hypertrophy (LVH), which normalises wall stress according to Laplace’s law. This is initially adaptive, but decompensation eventually occurs leading to the development of heart failure, symptoms and adverse events. Current clinical guidelines suggest valvular intervention in severe aortic stenosis when there is evidence of LV decompensation as indicated by the development of either symptoms or impaired LV ejection fraction (EF) [5, 6]. However, assessment of symptoms in elderly patients who often have multiple comorbidities can be challenging whilst impairment of LV systolic function occurs late in the disease process [7] and is often irreversible [8, 9]. There is therefore a need for more objective assessments of the left ventricular decompensation. Similarly, in aortic regurgitation, the left ventricle dilates in response to chronic volume overload in an eccentric hypertrophic response. With time, this decompensation of this remodelling response also occurs, leading to heart failure, symptoms and adverse events in the absence of treatment. Current guidelines advocate valve replacement in the presence of severe aortic regurgitation and symptoms or when LV dilatation reaches certain thresholds.

In this review, we will describe how modern advances in non-invasive imaging might optimise assessments of aortic valve stenosis and regurgitation as well as how the left ventricular remodels in response to those lesions. In particular, the established role of echocardiography will be explored alongside emerging modalities such as computed tomography (CT), cardiovascular magnetic resonance (CMR) and positron emission tomography (PET).

## Aortic Stenosis

### Echocardiography

Transthoracic echocardiography (TTE) is the clinical imaging modality of choice for assessing aortic stenosis and has been since in the 1980s when it supplanted invasive catheter-based measurements. It is safe, non-invasive and widely available, allowing direct visualisation of aortic valve anatomy (e.g. bicuspid vs. trileaflet), function and hemodynamics whilst also facilitating measurement of the left ventricular wall thickness, cavity dimensions and both systolic and diastolic function.

**Fig. 1:**
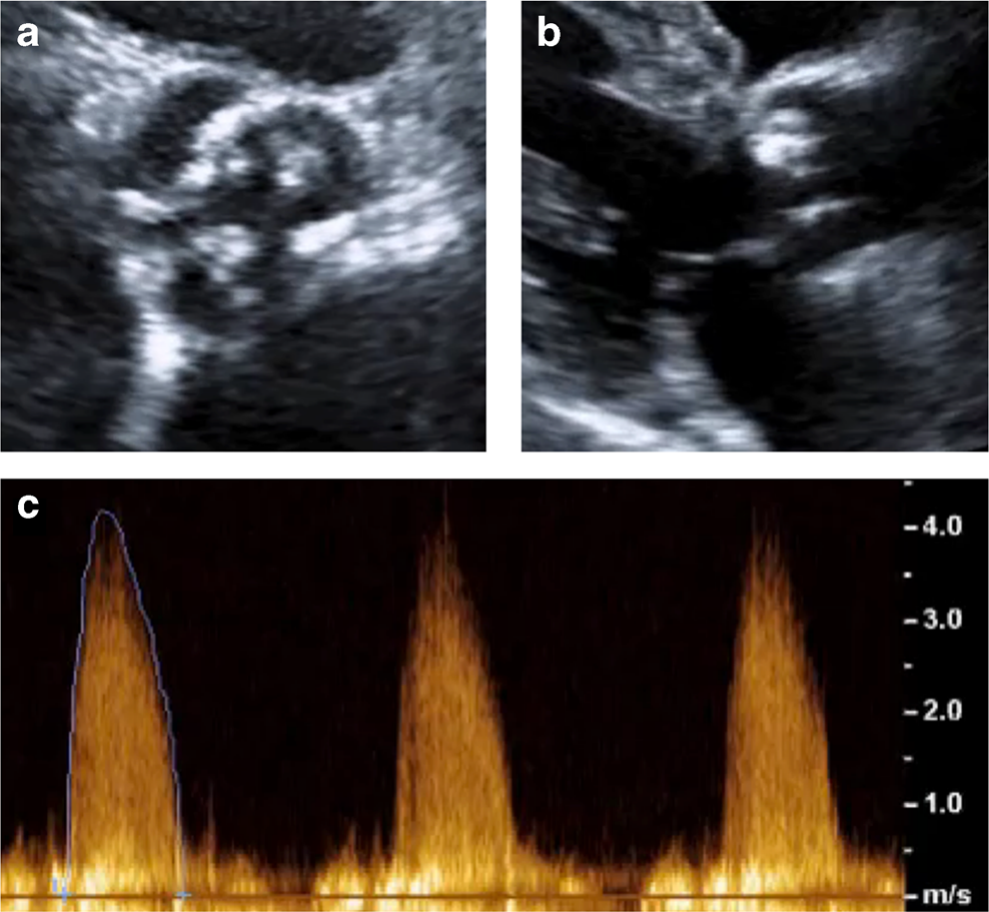
Echocardiographic assessment of a patient with severe aortic stenosis. **a** Short axis view showing heavily calcified leaflets. **b** Parasternal long axis view showing large calcium deposit on right coronary cusp with restricted valve opening. **c** Right sternal edge continuous-wave Doppler with aortic valve velocity >4 m/s, corresponding with severe stenosis

Doppler echocardiography provides information on aortic valve hemodynamics that is not readily available using other imaging modalities. Simple assessments of both peak and mean velocities through the aortic valve (Fig. 1) are used to calculate peak and mean pressure gradients using the modified Bernoulli formula as well as the aortic valve area (AVA) using the continuity equation. The latter is flow independent and therefore often essential for diagnostic accuracy particularly in low-flow states [10]. Current guidelines recommend grading haemodynamic severity of aortic stenosis on the basis of the combined information provided by the peak velocity (AV Vmax), the mean gradient and the aortic valve area [5, 6].

Whilst this combined approach is effective in the majority of patients, it leads to a wide spectrum of diagnostic categories and the potential for clinical confusion. Other potential limitations of echocardiography are also being increasingly appreciated. Firstly, acquisition of diagnostic acoustic windows can be impossible in certain patients as can perfect alignment of the Doppler probe with the direction of maximal blood flow through the valve. In both circumstances, measurement errors will be introduced. Secondly, echocardiography may have difficulty in measuring the left ventricular outflow tract (LVOT) diameter with accuracy: a key component when using the continuity equation to calculate the aortic valve area. Indeed, echo often underestimates the LVOT diameter due to either calcification or its elliptical shape, and as the measurement is squared, even small errors become magnified substantially. The continuity equation also relies on several geometric assumptions that frequently do not hold true in aortic stenosis (such as a circular outflow tract and laminar flow profile), introducing further error. Finally, internal inconsistencies exist in the severity thresholds established in the clinical guidelines. An AVA of 1.0 cm^2^ is sensitive but less specific for severe aortic stenosis and in fact corresponds to a mean pressure gradient of 30–35 mmHg [11], rather than the 40 mmHg cut-off recommended [5, 6]. This in part may explain why between 20 and 30 % of patients with moderate or severe aortic stenosis have discrepant assessments of disease severity depending on the echocardiographic marker assessed [4, 12].

#### LV Function and Mass

Echocardiography-derived LV ejection fraction is used in clinical guidelines to reflect LV systolic function. Impairment in the EF below 50 % is an indication for valve intervention as these patients have a poor outcome without surgery [8, 13]. However, a fall in ejection fraction is an insensitive measure of LV systolic dysfunction in the presence of concentric remodelling and hypertrophy. Indeed, approximately one third of patients with aortic stenosis and a normal EF have significant evidence of LV systolic impairment when assessed by other methods [14]. These alternative markers include global longitudinal strain measurements, which have been shown to be of prognostic importance in patients with severe aortic stenosis and a normal ejection fraction [15].

Patients with aortic stenosis invariably develop the left ventricular hypertrophy as the LV remodels to normalise wall stress. The degree to which this occurs is not well correlated to the haemodynamic severity of stenosis and is an independent predictor of outcomes [16, 17•]. The LV hypertrophic response should therefore be assessed separately. Concentric remodelling geometry [18] and severe LVH [19] have been associated with mortality following valve replacement whilst increased LV mass is associated with cardiovascular morbidity and mortality in patients with asymptomatic severe AS [17•]. Importantly, recent evidence from 1656 patients in the SEAS trial showed that LV mass index was an independent predictor of cardiovascular events and all-cause mortality [20••].

#### Low-Flow Low-Gradient Subtypes

The most challenging patients are those with discordant parameters of severity, most commonly characterised by a low AVA and low transvalvular gradient. As discussed, there are several possible explanations for this including measurement error and internal inconsistencies in guideline thresholds. However, in many patients, the discrepancy will not be due to error but instead reflect a low-flow status related to an array of different factors. Low flow is usually defined by a stroke volume (SV) index of <35 ml/m^2^ although this cut-off is somewhat arbitrary.

#### Classical Low-Flow Low-Gradient AS

In patients with severe aortic stenosis and LV systolic dysfunction, the stroke volume is low due to reduced myocardial contractility. As a consequence, the gradient generated over the aortic valve is relatively low (mean gradient <40 mmHg) but the valve area is small <1.0 cm^2^ (low-flow low gradient with reduced EF severe AS). It is important to differentiate this condition from “pseudo-severe AS,” where the ventricle is severely impaired due to an alternative pathology to the extent that it cannot generate sufficient flow to completely open the aortic valve. Low-dose dobutamine stress echocardiography (DSE), as recommended in clinical guidelines [5, 6], can differentiate between these; if the mean valve gradient increases to >40 mmHg (or AV Vmax >4 m/s) and valve area remains <1.0 cm^2^ with dobutamine stress, then severe AS has been identified. These patients have a relatively low operative mortality (5–7 % [21, 22]) and benefit from AVR [23].

#### Flow Reserve

Those patients who fail to increase their gradient with stress echocardiography likely have no or reduced “flow reserve” which is defined as an increase in stroke volume of less than 20 % [24]. This group of patients has significantly higher operative mortality (22–30 % [13, 21]), but those who survive AVR have outcomes (improvement in EF and mortality) similar to those with flow reserve [13, 25] and an improved prognosis compared to similar patients managed medically [13]. There may be an increased future role for transcatheter aortic valve implantation (TAVI) in this group given their high operative risk.

#### Paradoxical Low-Flow Low-Gradient AS

These patients have low flow in the context of preserved ejection fraction, again leading to a picture of a reduced AVA (<1.0 cm^2^) and low mean gradient (<40 mmHg). It is often referred to as low-flow low-gradient normal EF or paradoxical low-flow low-gradient aortic stenosis and was first identified in 2007 [26]. Commonly, these patients are female and elderly, with a small hypertrophied LV cavity as the cause of their low stroke volume. A recent meta-analysis of 7459 patients and other studies have indicated that mortality is increased in this group [26, 27••, 28, 29] and reduced by valve intervention [28–31]. However, this has not been observed consistently in all trials [32]. Stress echocardiography has not been shown to be helpful in these patients as they often exhibit restrictive physiology due to diastolic dysfunction limiting any increase in SV; however, aortic valve CT calcium scoring may aid in discrimination [12]. Current clinical guidelines recommend aortic valve intervention in this group if the patient is symptomatic and the clinician feels that valve obstruction is the most likely cause of symptoms based on the above parameters [5, 6].

#### Normal-Flow Low-Gradient AS

Patients with both a low AVA and low mean gradient in the context of preserved EF and normal flow are a common [27••] but under recognised group who are not represented in clinical guidelines. Although this is heterogeneous group that encompasses measurement errors, small body size or inconsistencies in clinical guidelines [11], a significant proportion have severe AS [12] and AVR appears to improve survival [31]. A recent large meta-analysis has demonstrated that these patients have outcomes similar to high-gradient severe AS which are improved by AVR [27••]. Further research in this area is required.

#### Dimensionless Index

The dimensionless velocity index is a flow-independent variable calculated by dividing the LVOT velocity-time integral (VTI, or Vmax) by the AV VTI (or Vmax) without a need to measure the LVOT diameter. A ratio of <0.25 indicates severe stenosis and is particularly useful where LVOT measurement is difficult to perform or in cases of inconsistent grading [33].

#### Advanced Echocardiography

In addition to demonstrating flow reserve in low-flow low-gradient severe AS with a reduced ejection fraction, stress echocardiography has also been shown to improve prognostication in asymptomatic high-gradient severe AS where an increase in mean gradient of >20 mmHg on exercise stress predicts a greater risk of developing symptoms and adverse events [34, 35].

Transoesophageal echocardiography (TOE) can be of use in aortic stenosis with planimetry of the AVA used as an alternative measure of aortic stenosis severity. Whilst planimetry remains difficult on 2D imaging due to extensive calcification and difficulty ensuring position at the leaflet tips, it appears more readily feasible on 3D TOE. A study of 307 patients with severe aortic stenosis compared valve planimetry using 3D TOE with TTE-derived aortic valve area. They showed that valve planimetry was possible in 92 % of patients (in the 8 % where it was not possible, this was due to severe calcification) and that the two measurements showed a good correlation (*r* = 0.85). However, planimetred AVA measurements were consistently higher than those calculated with the continuity equation [36]. Adjudicating disease severity using planimetry can therefore be difficult although in that context, an AVA <1.0 cm^2^ is a strong indication of severe aortic stenosis and a potentially useful arbitrator in cases of diagnostic uncertainty.

TOE also offers accurate assessment of the aortic root and annulus dimensions and is frequently performed preoperatively before aortic valve surgery. Similar measurements can be made with CT imaging and the modality used differs between centres. The use of intraoperative TOE is routine in many cardiothoracic centres where it allows accurate assessment of anatomy and optimisation of hemodynamics before establishing cardiopulmonary bypass. Post-procedure, TOE can confirm satisfactory valve function, stable hemodynamics and exclude complications such as outflow tract obstruction. A number of observational studies suggest that intraoperative TOE changes management in 11–18 % of patients may improve outcome [37, 38] and may be cost-effective [39]. Intraoperative TOE has a class lla recommendation from the most recent CC/AHA/ASE 2003 Guideline Update for the Clinical Application of Echocardiography.

Pre-procedural imaging (TOE or CT) is essential prior to TAVI to ensure correct prosthesis sizing, and real-time intra-procedural TOE is often used to aid in device sizing and positioning [40, 41], although this is limited to trans-apical and aortic approaches where the patient is under general anaesthetic. Studies are conflicting but suggest that there is overall a slight overestimation of annulus area with CT and underestimation with TOE [42, 43]. 3D TOE is superior to 2D TOE and offers similar results to CT in some studies [44].

#### Valvular Calcification

Although the mechanisms underlying valvular calcification remain incompletely determined [3], its importance to disease progression and adverse events was first identified in the seminal studies by Rosenhek and colleagues [45••, 46••]. One hundred and twenty-six patients with asymptomatic severe aortic stenosis were followed up for 22 ± 18 months. Aortic valve calcification was measured on a four-point ordinal scale with moderate or severe calcification (a score of 3 or 4) being the only independent predictor of AVR or mortality, outperforming haemodynamic measures of severity. Significant valve calcification is also associated with faster disease progression, need for AVR and all-cause mortality in patients with mild to moderate stenosis [46••]. Whilst severe aortic valve calcification is considered a lla indication for AVR in asymptomatic patients with severe AS, this technique is in practice difficult to apply because of poor intra-observer agreement as to the severity of calcification [47].

### CT

#### CT Calcium Scoring

Calcium burden in the aortic valve can be more accurately quantified on electrographically gated non-contrast computed tomography (CT). The aortic valve CT calcium score can then be measured using the Agatston score (AU), which accounts for both the density and volume of CT measured calcium and correlates closely with the weight of calcium in explanted aortic valves [47]. Aortic valve CT calcium scoring has demonstrated excellent intra- and inter-observer and scan-rescan reproducibility [47, 48] and correlates closely with echocardiographic measures of haemodynamic severity [47–49]. Importantly, recent data has demonstrated that the aortic valve CT calcium score provides powerful prediction of disease progression and prognosis [50–52].

#### Severity Cut-Offs

**Fig. 2:**
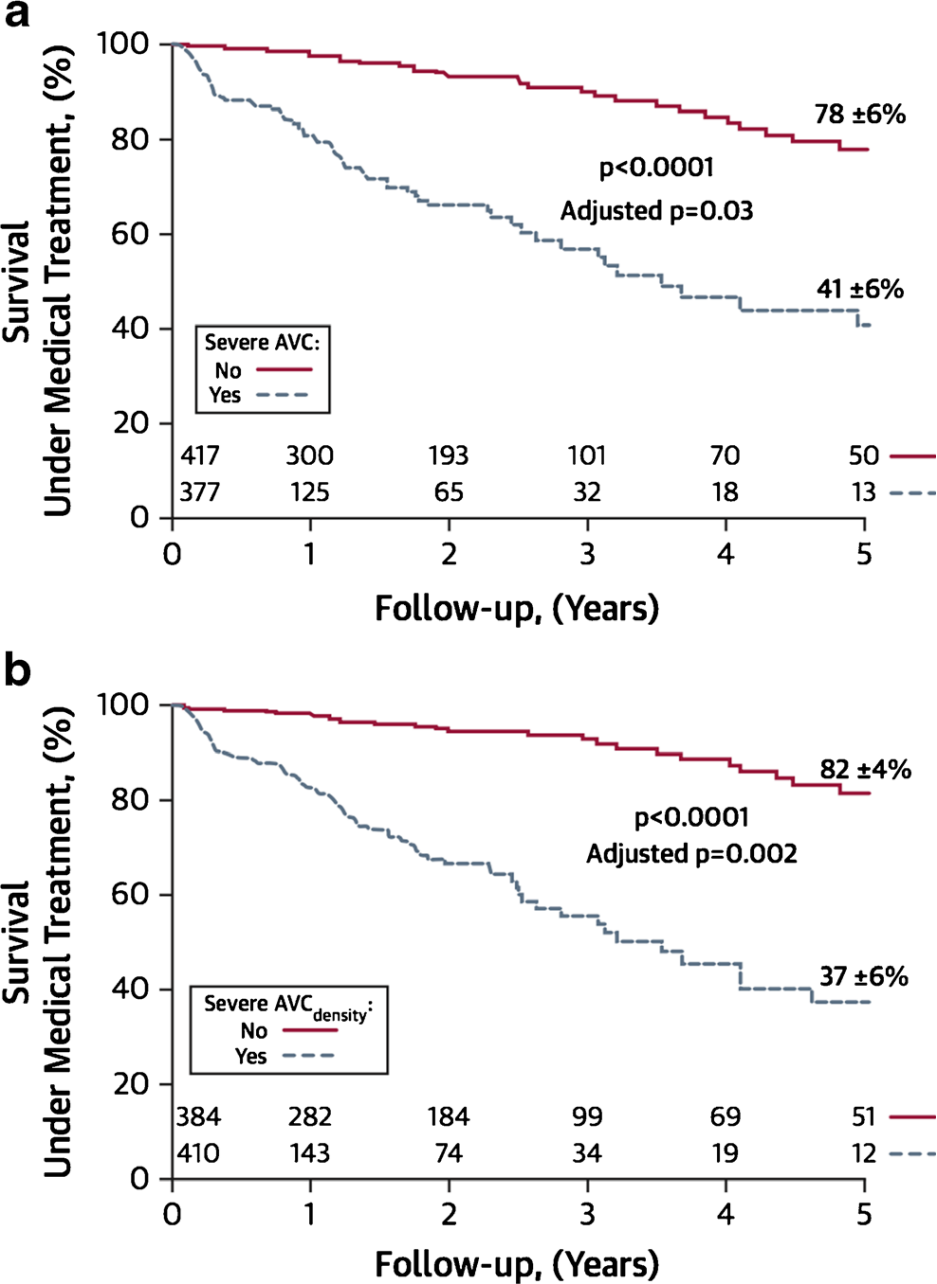
Survival of patients with aortic stenosis under medical treatment according to valvular calcium score. Patients with severe absolute calcification (**a**) or calcification indexed to body surface area (**b**) had increased all-cause mortality compared to patients with non-severe calcification. Indeed, severe aortic valve calcification (AVC) was an independent predictor of survival following adjustment for age, sex, presence of coronary artery disease or diabetes, indexed aortic valve area and ejection fraction. Reproduced from Clavel et al. [53••] with permission from Elsevier/Journal of the American College of Cardiology

Thresholds in CT calcium score for differentiating moderate from severe aortic stenosis have recently been proposed in a study of 451 patients with concordant grading of AS severity on echocardiography and preserved ejection fraction. Interestingly, these were different for males and females (≥2065 AU for men and ≥1274 AU for women) even after indexing to the aortic annulus area (≥476 AU/cm^2^ for men and ≥292 AU/cm^2^ for women). These thresholds were then applied to a larger cohort of 794 patients and demonstrated a strong predictive value for all-cause mortality of incremental value to echocardiographic parameters of ejection fraction and stenosis severity [53••] (Fig. 2).

Aortic valve calcium scoring may be of particular use in cases of low-flow low gradient with reduced EF [49, 54], especially in the absence of flow reserve [47] where it can be challenging to determine severity by echocardiography alone. Further work is required to assess the validity of these thresholds in alternative patient populations and to confirm their predictive value. If these prove confirmatory, then we believe CT calcium scoring will emerge as a clinically useful and flow-independent adjuvant to standard echocardiography.

#### Improved AVA Calculation

The increasing use of CT angiography for valve sizing prior to TAVI procedures has demonstrated that the LVOT is often eccentric not circular. Indeed, a recent study of 269 patients with severe AS undergoing CT demonstrated that the LVOT is eccentric in 93 % of patients [55]. As a consequence, TTE measures of the LVOT diameter can frequently result in underestimation of the true AVA [56]. Using CT, CMR or indeed 3D echo to planimeter the LVOT area could therefore improve the accuracy of AVA calculations.

### PET

PET is a novel imaging technique, which allows the activity of specific disease processes to be measured in vivo. Recently, this technique has employed two tracers to measure inflammation (^18^F-fluorodeoxyglucose (^18^F-FDG)) and calcification activity (^18^F-fluoride) in the valves of patients with aortic stenosis. Hybrid PET/CT scanners then allow the activity of these two key processes to be compared with the presence of established regions of macrocalcification on CT.

#### ^18^F-Fluoride

**Fig. 3:**
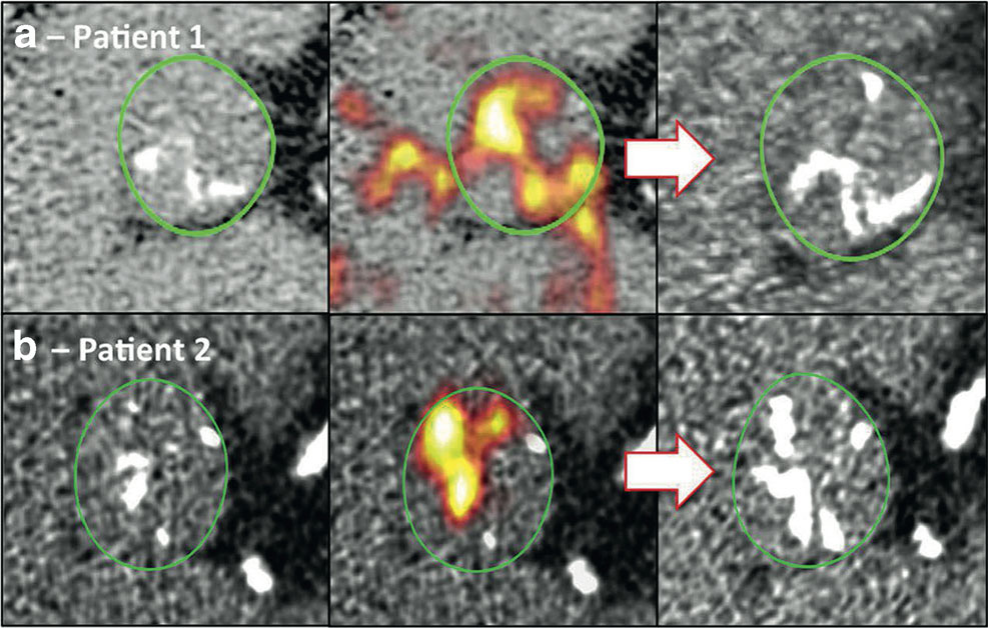
18F-fluoride PET activity predicts the development of new calcific deposits in the aortic valve on repeat CT imaging performed after 1 year. Example imaging from two patients (a and b) are shown below. Baseline non-contrast CT images (*left*) showed evidence of increased 18F-Fluoride PET activity (*middle*) in areas where subsequent calcification was observed on repeat CT scanning after 1 year (*right*). Reproduced from Pawade et al. [3] with permission from Elsevier/Journal of the American College of Cardiology

^18^F-fluoride has been used as a bone tracer for 50 years binding to hydroxyapatite crystal and detecting regions of increased bone activity. In the vasculature, it binds preferentially to regions of newly developing microcalcification because the surface area of hydroxyapatite in these nanocrystalline regions is at its highest. By contrast in regions of macrocalcification, much of the hydroxyapatite is internalised and not available for binding [57]. In aortic stenosis, ^18^F-fluoride acts as a marker of calcification activity correlating with histological staining for alkaline phosphatase (*r* = 0.65) and osteocalcin (*r* = 0.68) [52] and predicts where novel regions of macroscopic calcium are going to form (Fig. 3). Tracer uptake increases with more advanced aortic stenosis [58], offers powerful prediction of disease progression at 1 and 2 years, of small incremental value to computed tomography [52, 59], and acts as an independent predictor of adverse clinical events [59]. This technique holds promise in better understanding the role of calcification in aortic stenosis, for example, a recent PET study demonstrated that whilst calcification activity in aortic stenosis is greater than inflammation, the reverse is true in atherosclerosis, potentially explaining the different effects of statins in these two conditions [60]. With further improvement, ^18^F-fluoride PET may prove of clinical use in identifying patients likely to progress rapidly towards surgery and as a marker of disease activity and efficacy end point in clinical trials of novel therapies (e.g. SALTIRE 2: NCT02132026).

#### ^18^F-FDG

^18^F-FDG PET is widely used to image vascular inflammation. This PET tracer is a glucose analogue, which accumulates in metabolically active cells such as vascular macrophages. Indeed, an excellent correlation between macrophage burden on histology (CD68 staining on immunohistochemistry) in carotid atheroma [61] and the ^18^F-FDG signal has been observed. In aortic stenosis, ^18^F-FDG activity is higher in patients versus controls, demonstrating a modest correlation with severity of valvular disease [58]. Of interest, no correlation with CD68 staining of explanted valves was observed suggesting that ^18^F-FDG uptake is occurring in other metabolically active cells, although this study was limited by a low sample size [52]. Perhaps, the biggest limitation of this technique is the effect of physiological myocardial ^18^F-FDG uptake, which frequently contaminates signal originating from the aortic valve.

### CMR

Cardiac magnetic resonance is an emerging technology that offers excellent spatial resolution, functional assessment and the unique ability to provide myocardial tissue characterisation. However, it remains an expensive modality with limited availability for cardiac patients in most centres,

#### LV Mass and Hypertrophy

CMR provides the gold-standard assessment of LV volumes and mass and allows detailed investigation of both the degree of hypertrophy and the different patterns of the left ventricular adaption. Importantly, the myocardial hypertrophic response is only weakly correlated with the hemodynamic severity of aortic stenosis [16, 62, 63], with males generally display a greater increase in LV mass even after correction for body size [16]. Classically, wall thickening occurs in a concentric pattern, but recent studies have shown that asymmetrical patterns also exist in around a quarter of patients assessed by CMR [16]. The clinical importance of this observation remains unclear.

#### Myocardial Fibrosis

**Fig. 4:**
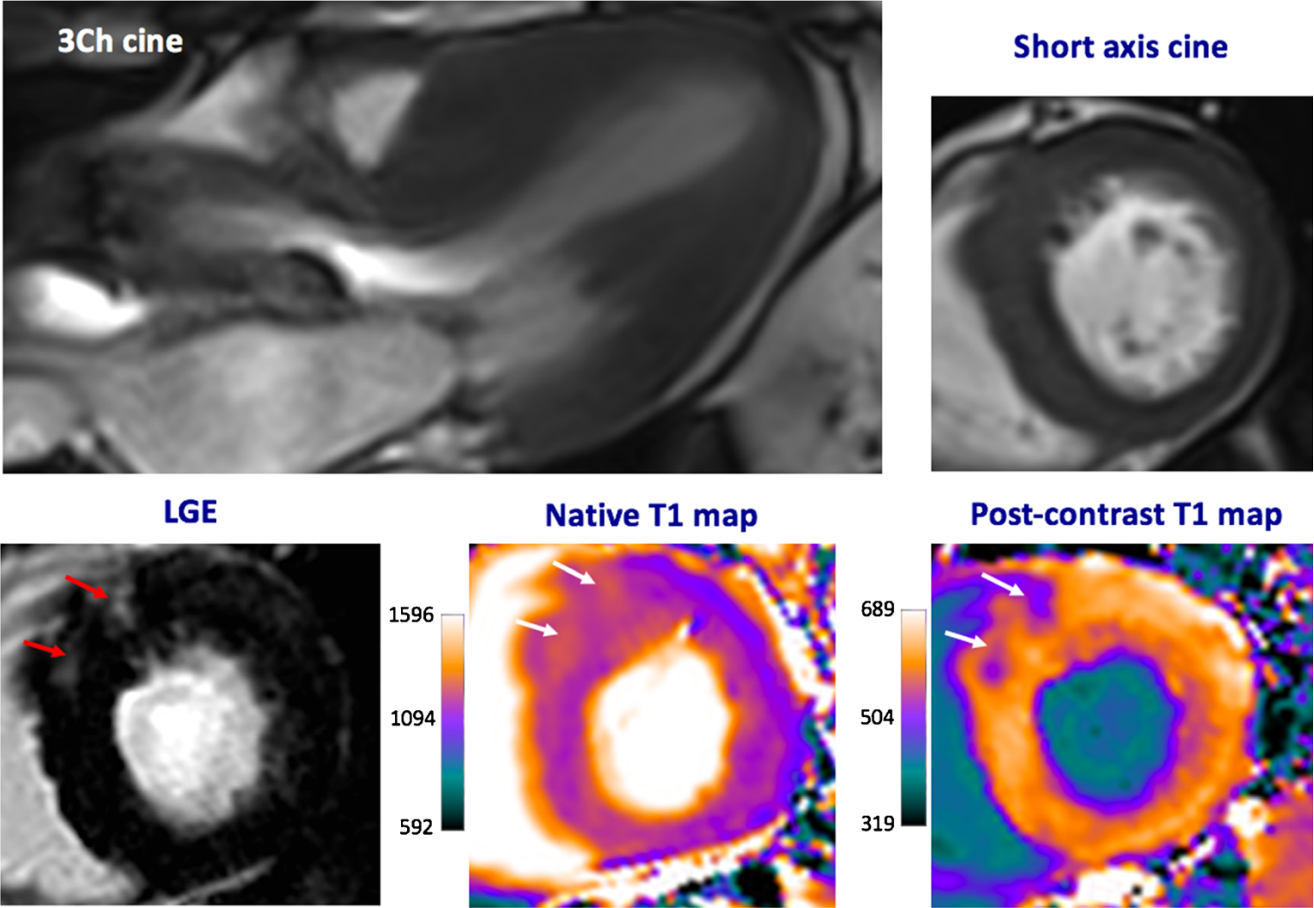
Cardiac magnetic resonance imaging in a patient with severe aortic stenosis. Predominant asymmetrical hypertrophy of the anteroseptum is seen with associated patchy mid-wall late gadolinium enhancement (LGE, *red arrows*). These areas are also identified visually using native and post-contrast T1 maps (*white arrows*)

Myocardial fibrosis is a key mechanism driving the progression from the left ventricular hypertrophy to heart failure and death in aortic stenosis [64]. Historically, it has only been appreciated using invasive endomyocardial biopsy techniques, but this carries a small but significant risk of complications [65] and is susceptible to sampling error. CMR provides a non-invasive assessment of whole-heart fibrosis using two techniques: late gadolinium enhancement (LGE) and T1 mapping (Fig. 4).

#### LGE

This technique was first described in 1999 [66] and involves the intravenous administration of gadolinium-based contrast agents (GBCA). These agents alter myocardial T1 values and enter healthy myocardium from the blood pool down a concentration gradient within 1–3 min (wash-in phase). Renal excretion of GBCA from the blood pool then produces a reverse concentration gradient with myocardial GBCA concentrations declining over the ensuing 10–30 min (wash-out phase). The large molecular size of gadolinium stops GBCA from crossing cell membranes, so that they effectively label the extracellular space and accumulate in regions of replacement fibrosis due to delayed wash-out [67]. These focal areas can then be detected using T1-weighted sequences 15–20 min after contrast administration. In aortic stenosis, areas of replacement fibrosis appear as bright areas in the mid-wall of the left ventricle in contrast to surrounding healthy myocardium [68]. Areas of previous myocardial infarction, which are common in AS patients, are also detected by this technique but can be differentiated from mid-wall replacement fibrosis by their subendocardial/transmural pattern and their coronary distribution.

Mid-wall replacement fibrosis as detected by LGE is common in aortic stenosis (29–62 % of patients depending on the population studied [69, 70•, 71•]) and seems to be irreversible following valve intervention [72]. The presence of LGE correlates with histological fibrosis [73•] and evidence of myocardial injury (as measured by high sensitivity troponin I concentrations) [74]. Advanced mid-wall fibrosis identifies patients that do not gain improvement in LV systolic function [73•] or overall functional status following AVR [72]. Importantly, three studies have confirmed that the presence of LGE acts as an independent predictor of all-cause mortality [70•, 71•, 73•], increasing the risk of death up to eightfold [70•]. Mid-wall fibrosis therefore appears to be a direct marker of the left ventricular decompensation in aortic stenosis and may be of use in identifying patients whose ventricle are starting to fail and who might benefit from prompt AVR. Further research on this area is needed; indeed, EVoLVeD-AS a multicentre randomised-controlled trial assessing the benefit of early surgery in patients with advanced aortic stenosis and mid-wall fibrosis on CMR is due to the start enrolling patients next year.

#### Diffuse Fibrosis

The non-invasive assessment of diffuse fibrosis is more challenging. Its homogeneous nature means that it is missed on LGE techniques, which rely on regions of normal myocardium to generate contrast. However, the detection of diffuse fibrosis is important because it is widely believed to be reversible [75] and the precursor to irreversible forms of replacement fibrosis.

Myocardial T1 mapping techniques enable the calculation of a specific T1 relaxation time (native T1) for each CMR voxel which can then be displayed on a 2D map with colour overlays applied for easier visual analysis. Multiple different techniques have been developed (Table 1). Full examination of these techniques is beyond the scope of this article, but further information can be found in this recent review by Moon et al. [76]. In brief, *native T1 measurements* can be made without the need for contrast, an important potential advantage especially in subjects with severe renal dysfunction who are at risk of contrast-induced nephrogenic systemic fibrosis (NSF) [77]. GBCA can also be used to generate *post-contrast T1 maps* as gadolinium shortens T1 relaxation times. In principle, these images provide greater signal but they are influenced by individual variation in gadolinium kinetics and have suffered from poor reproducibility when studied in AS populations [78•]. Importantly, these variations in kinetics can be corrected using several approaches. The partition coefficient (λ) is calculated as a ratio of myocardial to blood post-contrast T1 values, which improves reproducibility and corrects for many confounders. At gadolinium contrast equilibrium, the contrast concentration in the blood and myocardium should be equal. Calculating the blood volume of distribution (1—haematocrit) enables the myocardial volume of distribution to be deduced, also termed the extracellular volume fraction (ECV). Because ECV predominantly comprises collagen and is increased in fibrotic states, it acts as a marker of myocardial fibrosis, correlating closely with the collagen volume fraction on histology [79–82].

**Table 1:** T1 mapping measures available for assessment of myocardial fibrosis

Measure	Unit	Calculation	Advantages	Limitations
Native T1	ms	T1 relaxation curve	No gadolinium requirement (can use in severe renal failure)	T1 signal represented a composite of myocardium and extracellular space
Post-contrast T1	ms	T1 relaxation curve following gadolinium administration	Improved sensitivity in identifying myocardial fibrosis	Significant variability due to individual variation in gadolinium kinetics and time to imaging post-contrast injection
Partition coefficient (λ)	Ratio	Ratio of T1 signal change (pre- and post-contrast) in myocardium and blood pool	Excellent scan-rescan reproducibility	Does not account for plasma volume of distribution of gadolinium contrast
Extracellular volume fraction (ECV)	%	ECV = λ × (1—haematocrit)	Excellent scan-rescan reproducibility. Conceptually attractive measure	Gives a measure of relative fibrosis which may not best track changes in aortic stenosis
Fibrosis volume	ml	ECV × end-diastolic volume	Quantitative measure of absolute fibrosis volume	Limited evidence at current time May require indexing to body size to enable comparison between individuals

Although native T1 and ECV have been extensively studied in the literature, results are mixed and interpretation is confounded by heterogenous studied populations, variations in T1 mapping sequence, CMR scanner, magnetic field strength and analytical technique (e.g. inclusion or exclusion of areas of LGE).

Native T1 values appear to correlate with histological myocardial fibrosis [83, 84] as well as global longitudinal strain [84], LV mass, haemodynamic assessments of severity and patient functional status [83]. However, its ability to differentiate healthy patients from controls is dependent on the population studied [78•, 83] with a significant degree of overlap in T1 values between these groups particularly subjects with less advanced stenosis. In a population of non-ischaemic dilated cardiomyopathy patients, native T1 has recently been shown to be an independent predictor of all-cause mortality and heart failure events [85] although this has not been demonstrated in aortic stenosis patients.

#### ECV

The extracellular volume fraction is used as a surrogate for the extracellular space, which is expanded with collagen deposition in diffuse fibrosis. Our centre has demonstrated superior intra- and inter-observer and scan-rescan variability in aortic stenosis compared to the other T1 measures, and ECV correlates with LV diastolic dysfunction [78•] and functional status [86]. However, prognostic data is currently lacking. There is also significant overlap between ECV values obtained in healthy volunteers and AS patients, and normal ECV values have been observed in a hypertensive population (another condition characterised by LV pressure overload) [87].

Another disadvantage is that ECV measures fibrosis relative to the volume (or mass) of the left ventricle. Balanced increases in both LV mass and diffuse fibrosis with progressive aortic stenosis are therefore not detected using this approach. In fact, an important study by Krayenbeuhl et al. involving serial myocardial biopsies demonstrated that histological myocardial fibrosis as a percentage of the myocardium (which is estimated by ECV calculation on CMR) actually increased early following aortic valve surgery as a result of significant reduction in LV mass with no change in the amount of fibrosis. However, the overall fibrous content (which can be estimated on CMR by fibrosis volume; ECV × end-diastolic myocardial volume) did eventually decrease at a later stage (repeat biopsy an average of 70 months post AVR) [75]. This is partly supported by a recent CMR study which found that ECV did not change at 6 months following AVR, whereas there was significant regression of cellular hypertrophy [86]. There is however no CMR data regarding late regression of diffuse fibrosis measures. We believe the use of the fibrosis volume as a measure of absolute fibrosis may better reflect disease progression and be able to track changes across interval scans, although this requires investigation in prospective studies.

#### Clinical Risk Score

CMR is an expensive technique with limited availability in many centres. We have devised a clinical risk score [88] based on five readily measured variables: age, sex, peak aortic valve velocity, high sensitivity troponin I concentration and presence of LV strain pattern on ECG, which is highly predictive of the presence of mid-wall replacement fibrosis on CMR and mortality. Ultimately, this could be used clinically in place of CMR imaging or as a screening tool for LV decompensation in aortic stenosis.

#### Valve Assessment

CT imaging is able to detect macroscopic calcium deposits in the aortic valve but is unable to identify fibrosis or lipid deposition, which are key components in the stenotic valve. CMR offers superior tissue characterisation as demonstrated in a proof of concept study where 30 explanted aortic valves were scanned *ex-vivo* and compared with histological analysis. CMR showed excellent sensitivity and specificity for the identification of both mineralisation (calcification) and fibrosis, with lower accuracy for lipid-rich tissues [89]. Although this is an exciting field for further research, in vivo imaging with this approach is not currently feasible due to leaflet motion.

## Aortic Regurgitation

### Echocardiography

TTE remains the first-line imaging modality in the investigation of patients with aortic regurgitation, allowing assessment of mechanism, valve morphology and severity of regurgitation as well as measures of LV remodelling and function. Imaging of the aortic root and ascending aorta is essential, although in patients with poor acoustic windows, cross-sectional imaging may be required for accurate assessment.

The assessment of aortic regurgitation severity is more nuanced than aortic stenosis, requiring the integration of different visual, semi-quantitative and quantitative parameters as recommended by clinical guidelines [5, 6]. Visual assessment of the valve leaflets allows appreciation of prolapse or non-coaptation, whilst the length and width of the regurgitant jet on colour Doppler gives a qualitative impression of severity. Whilst useful, these measures correlate only modestly with the following more objective measures of AR severity which also require assessment [90].

#### Semiquantitative Parameters

Calculating the ratio of the regurgitant jet width to that of the LVOT gives a semiquantitative measure of AR severity (severe if >65 %) [91]. The vena contracta (the narrowest part of the regurgitant jet) can also be measured, and a width of >0.6 cm suggests severe AR. Both these techniques are limited by a single plane of assessment and the assumption of a circular regurgitant orifice. 3D TTE may therefore allow more accurate measurements [92].

#### Doppler-Based Measures

Although continuous wave AR Doppler jet density is a poor marker of severity, the rate of deceleration (pressure half-time, PHT) is a useful adjunct to other measures. A value of <200 ms is considered severe with measurements critically dependent on obtaining an aligned Doppler signal. PHT is best used in addition to other parameters as it is affected by LV compliance, blood pressure and usually reduced in acute AR of any severity. Doppler assessment of aortic flow direction is highly useful where imaging windows allow. Holodiastolic flow reversal in the descending aorta, especially when associated with an end-diastolic velocity of >20 cm/s, is specific but not sensitive for severe AR [93].

#### Quantitative Parameters

Calculation of effective regurgitant orifice area (EROA) or regurgitant volume (RV) is possible in some patients using the flow-convergence zone (PISA) method, which is less sensitive to loading conditions than other measures and also useful if the jet is eccentric. It is however less well studied than in mitral regurgitation, assumes a circular orifice (with a hemispheric flow convergence zone) and is impossible to measure in a substantial proportion of patients [90]. An alternative is the regurgitant fraction (RF), which can be calculated by the Doppler volumetric method. This involves comparing the systemic stroke volume (calculated by assessing the flow over either the mitral or pulmonary valves assuming no significant valvular regurgitation) with the total stroke volume (calculated from LVOT flow.) This is time-consuming and the potential for compounding multiple small measurement errors can lead to substantial overall inaccuracies. Again, the use of 3D TTE may be superior in calculating regurgitant fraction [94].

#### LV Dimensions and Function

The response of the LV to chronic volume overload is chamber dilatation and hypertrophy. Left ventricular end-systolic diameter (LVESd) is an independent predictor of the development of cardiac symptoms or LV dysfunction [95, 96] and the risk of progression or mortality approaches 20 % when LVESd >5.0 cm [97]. LV systolic impairment occurs late in the disease process and is associated with poor prognosis [98] which is improved following AVR [99]. Current clinical guidelines advise valve intervention for asymptomatic severe AR in the presence of significant LV dilatation (LVESd >5.0 cm) or LV systolic impairment [5, 6]. Other measures of LV function such as global strain and strain rate may detect earlier decompensation, and further research on outcomes is needed [100–103].

#### TOE

As with aortic valve replacement for aortic stenosis, transoesophageal echocardiography is frequently used (with significant variation between centres) both pre- and intraoperatively to aid in prosthesis sizing, confirm satisfactory prosthesis functioning and detect immediate post-operative complications. In centres with appropriate expertise, TOE also allows detailed assessment of valve morphology permitting valve preserving repair procedures in selected patients, particularly those with aortic root aneurysms or regurgitant non-calcified bicuspid valves [104].

### CMR

**Fig. 5:**
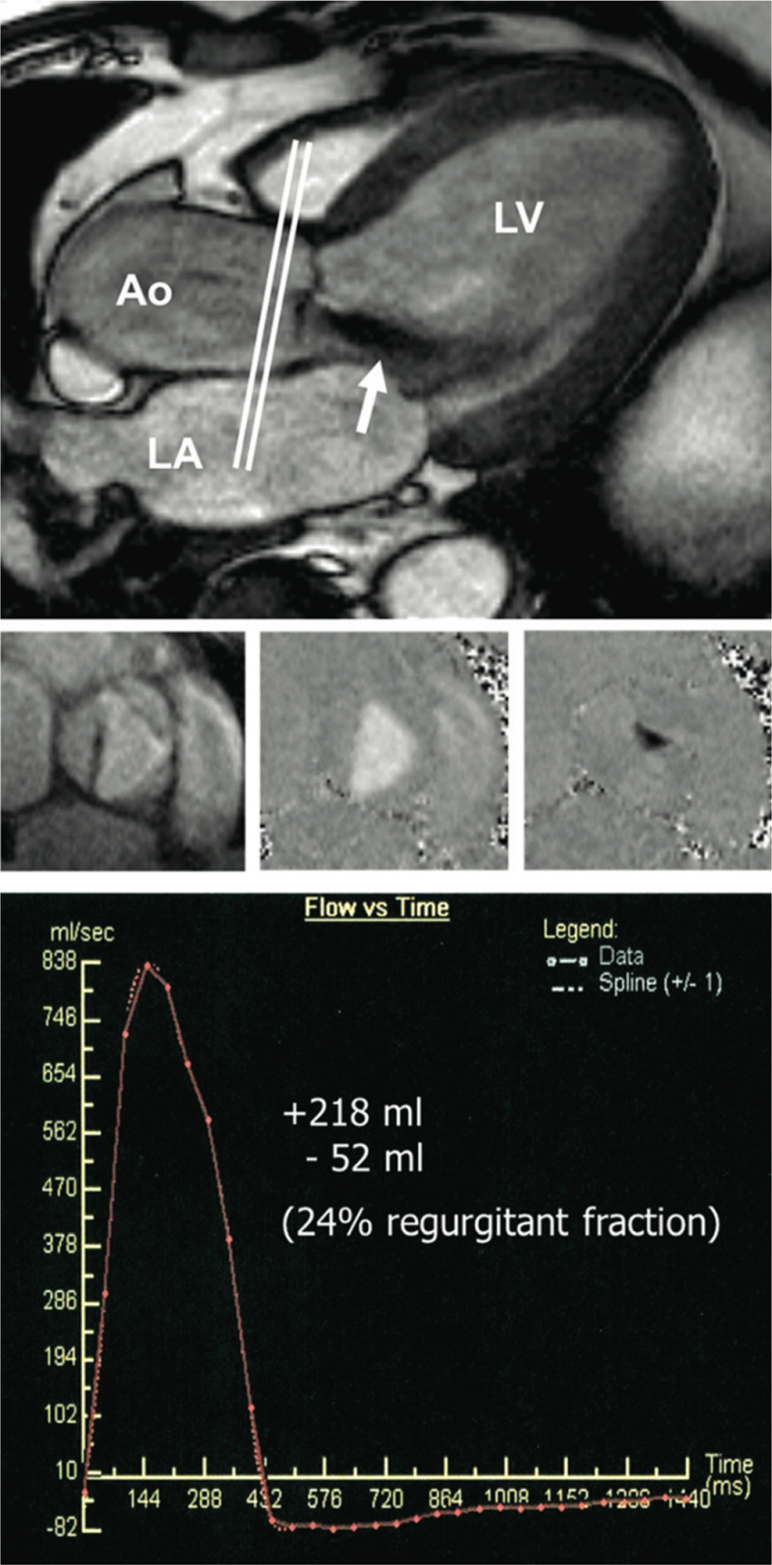
Phase-contrast velocity mapping for aortic regurgitation quantification. The slice location for through plane measurement is shown on a three chamber still image (*top*) with a jet of aortic regurgitation visible (*white arrow*). Through plane images are shown (*middle*) in systole depicting magnitude (*left*) and flow (*middle*) and diastole showing regurgitation in black (*right*). Regurgitant volume and fraction can then be calculated from a time-flow curve (*bottom*). *LV* left ventricle, *Ao* aorta, *LA* left atrium. Reproduced from Myerson et al. [106••] with permission from Wolters Kluwer Health, Inc./Circulation

As discussed, CMR provides the gold-standard assessment of LV volumes and ejection fraction [105]. Perhaps unsurprisingly, therefore, left ventricular dilatation detected by CMR (end-diastolic volume (EDV) >246 ml) has shown strong predictive ability for the future development of symptoms and need for valve surgery in AR [106••]. However, CMR is also able to determine the aortic regurgitant volume to a high degree of accuracy using phase-contrast velocity mapping. This map, created in an orthogonal plane to that of aortic flow (usually at the level of the sinotubular junction [107]), encodes flow to each voxel and covers the whole cardiac cycle. It can therefore be used to calculate both anterograde and retrograde flow (and ultimately regurgitant volume and fraction, Fig. 5). It shows superior reproducibility to echocardiography [108] and excellent correlation with both TTE assessment [109] and invasive measures of stroke volume [110]. There is some debate as to the optimal cut-off in the regurgitant fraction to define severe regurgitation. A value of 50 % as used in TTE would seem logical, but there is evidence of superior discrimination at a lower value of 30 % [111] and a RF above 33 % strongly predicted the need for surgery within 3 years in a series of 113 patients [106••]. Although there are some technical reasons why a discrepancy may exist, further work is required to corroborate this single centre study and to demonstrate improvement in patient outcomes using this more expensive imaging modality. However, there may be a place for CMR assessment of aortic regurgitation in clinical practice when there is diagnostic uncertainty as to severity of regurgitation.

## Conclusions

Aortic valve imaging is a rapidly expanding and exciting field. Although transthoracic echocardiography has limitations, it remains the first-line imaging modality of choice. However, other techniques are emerging which provide complimentary information and may aid clinical decision-making. In particular, CT can quantify the calcium burden in aortic stenosis as an alternative measure of disease severity. CMR can quantify the aortic regurgitant volume and provide detailed assessment of the hypertrophic response whilst PET can directly measure disease activity in the valve. Further research is required to investigate the role that these approaches may play in the future, where incremental clinical benefit to standard echocardiographic approaches will need to be demonstrated.

## References

[1] Nkomo VT, Gardin JM, Skelton TN, Gottdiener JS, Scott CG, Enriquez-Sarano M (2006). Burden of valvular heart diseases: a population-based study. Lancet.

[2] Carabello BA. Introduction to aortic stenosis. Circulation Research. Lippincott Williams & Wilkins; 2013;113:179–85. 10.1161/CIRCRESAHA.113.300156 23833292

[3] Pawade TA, Newby DE, Dweck MR (2015). Calcification in aortic stenosis: the skeleton key. J Am Coll Cardiol.

[4] Dweck MR, Boon NA, Newby DE. Calcific aortic stenosis. JACC. Elsevier Inc; 2012;60:1854–63. 10.1016/j.jacc.2012.02.093 23062541

[5] Joint task force on the management of valvular heart disease of the European Society of Cardiology (ESC), European Association for Cardio-Thoracic Surgery (EACTS), Vahanian A, Alfieri O, Andreotti F, Antunes MJ, et al. Guidelines on the management of valvular heart disease (version 2012). European Heart Journal. The Oxford University Press; 2012. pp. 2451–96.

[6] Nishimura RA, Otto CM, Bonow RO, Carabello BA, Erwin JP, Guyton RA, et al. 2014 AHA/ACC guideline for the management of patients with valvular heart disease: executive summary: a report of the American College of Cardiology/American Heart Association Task Force on Practice Guidelines. Circulation. Lippincott Williams & Wilkins; 2014. pp. 2440–92. 10.1161/CIR.0000000000000029 24589852

[7] Lancellotti P, Donal E, Magne J, Moonen M, O’Connor K, Daubert J-C, et al. Risk stratification in asymptomatic moderate to severe aortic stenosis: the importance of the valvular, arterial and ventricular interplay. Br Heart J. BMJ Publishing Group Ltd and British Cardiovascular Society; 2010;96:1364–71. 10.1136/hrt.2009.190942 20483891

[8] Connolly HM, Oh JK, Orszulak TA, Osborn SL, Roger VL, Hodge DO (1997). Aortic valve replacement for aortic stenosis with severe left ventricular dysfunction. Prognostic indicators. Circulation.

[9] Connolly HM, Oh JK, Schaff HV, Roger VL, Osborn SL, Hodge DO (2000). Severe aortic stenosis with low transvalvular gradient and severe left ventricular dysfunction:result of aortic valve replacement in 52 patients. Circulation.

[10] Rask LP, Karp KH, Eriksson NP (1996). Flow dependence of the aortic valve area in patients with aortic stenosis: assessment by application of the continuity equation. J Am Soc Echocardiogr.

[11] Minners J, Allgeier M, Gohlke-Baerwolf C, Kienzle R-P, Neumann F-J, Jander N. Inconsistent grading of aortic valve stenosis by current guidelines: haemodynamic studies in patients with apparently normal left ventricular function. Heart. BMJ Publishing Group Ltd and British Cardiovascular Society; 2010;96:1463–8. 10.1136/hrt.2009.181982 20813727

[12] Clavel M-A, Messika-Zeitoun D, Pibarot P, Aggarwal SR, Malouf J, Araoz PA (2013). The complex nature of discordant severe calcified aortic valve disease grading: new insights from combined Doppler echocardiographic and computed tomographic study. J Am Coll Cardiol.

[13] Tribouilloy C, Levy F, Rusinaru D, Guéret P, Petit-Eisenmann H, Baleynaud S (2009). Outcome after aortic valve replacement for low-flow/low-gradient aortic stenosis without contractile reserve on dobutamine stress echocardiography. J Am Coll Cardiol.

[14] Cramariuc D, Cioffi G, Rieck AE, Devereux RB, Staal EM, Ray S (2009). Low-flow aortic stenosis in asymptomatic patients: valvular-arterial impedance and systolic function from the SEAS Substudy. JACC Cardiovasc Imaging.

[15] Kusunose K, Goodman A, Parikh R, Barr T, Agarwal S, Popovic ZB (2014). Incremental prognostic value of left ventricular global longitudinal strain in patients with aortic stenosis and preserved ejection fraction. Circ Cardiovasc Imaging.

[16] Dweck MR, Joshi S, Murigu T, Gulati A, Alpendurada F, Jabbour A (2012). Left ventricular remodeling and hypertrophy in patients with aortic stenosis: insights from cardiovascular magnetic resonance. J Cardiovasc Magn Reson.

[17.•] Cioffi G, Faggiano P, Vizzardi E, Tarantini L, Cramariuc D, Gerdts E, et al. Prognostic effect of inappropriately high left ventricular mass in asymptomatic severe aortic stenosis. Heart. BMJ Publishing Group Ltd and British Cardiovascular Society; 2011;97:301–7. **This is the first paper suggesting that increased LV mass is an independent predictor of outcome in aortic stenosis**. 10.1136/hrt.2010.192997 20720251

[18] Duncan AI, Lowe BS, Garcia MJ, Xu M, Gillinov AM, Mihaljevic T (2008). Influence of concentric left ventricular remodeling on early mortality after aortic valve replacement. Ann Thorac Surg.

[19] Orsinelli DA, Aurigemma GP, Battista S, Krendel S, Gaasch WH (1993). Left ventricular hypertrophy and mortality after aortic valve replacement for aortic stenosis. A high risk subgroup identified by preoperative relative wall thickness. JACC.

[20.••] Gerdts E, Rossebø AB, Pedersen TR, Cioffi G, Lønnebakken MT, Cramariuc D, et al. Relation of left ventricular mass to prognosis in initially asymptomatic mild to moderate aortic valve stenosis. Circ Cardiovasc Imaging. 2015;8:e003644. **This large prospective study showed that increased LV mass is a predictor of cardiovascular event and all-cause mortality independent of age, sex, ejection fraction and presence of hypertension**. 10.1161/CIRCIMAGING.115.003644 PMC4648185 26489804

[21] Monin J-L, Quéré J-P, Monchi M, Petit H, Baleynaud S, Chauvel C, et al. Low-gradient aortic stenosis: operative risk stratification and predictors for long-term outcome: a multicenter study using dobutamine stress hemodynamics. Circulation. Lippincott Williams & Wilkins; 2003;108:319–24. 10.1161/01.CIR.0000079171.43055.46 12835219

[22] Nishimura RA, Grantham JA, Connolly HM, Schaff HV, Higano ST, Holmes DR (2002). Low-output, low-gradient aortic stenosis in patients with depressed left ventricular systolic function: the clinical utility of the dobutamine challenge in the catheterization laboratory. Circulation.

[23] Levy F, Laurent M, Monin J-L, Maillet JM, Pasquet A, Le Tourneau T (2008). Aortic valve replacement for low-flow/low-gradient aortic stenosis operative risk stratification and long-term outcome: a European multicenter study. J Am Coll Cardiol.

[24] de Filippi CR, Willett DL, Brickner ME, Appleton CP, Yancy CW, Eichhorn EJ (1995). Usefulness of dobutamine echocardiography in distinguishing severe from nonsevere valvular aortic stenosis in patients with depressed left ventricular function and low transvalvular gradients. Am J Cardiol.

[25] Quéré J-P, Monin J-L, Levy F, Petit H, Baleynaud S, Chauvel C, et al. Influence of preoperative left ventricular contractile reserve on postoperative ejection fraction in low-gradient aortic stenosis. Circulation. Lippincott Williams & Wilkins; 2006;113:1738–44. 10.1161/CIRCULATIONAHA.105.568824 16585393

[26] Hachicha Z, Dumesnil JG, Bogaty P, Pibarot P. Paradoxical low-flow, low-gradient severe aortic stenosis despite preserved ejection fraction is associated with higher afterload and reduced survival. Circulation. Lippincott Williams & Wilkins; 2007;115:2856–64. 10.1161/CIRCULATIONAHA.106.668681 17533183

[27.••] Dayan V, Vignolo G, Magne J, Clavel M-A, Mohty D, Pibarot P. Outcome and impact of aortic valve replacement in patients with preserved LVEF and low-gradient aortic stenosis. J Am Coll Cardiol. 2015;66:2594–603. **This meta-analysis provides the strongest evidence to date of increased mortality and survival benefit of AVR in patients with low-flow low-gradient severe aortic stenosis. Similar findings in patients with normal-flow low-gradient severe aortic stenosis are novel and require further research**. 10.1016/j.jacc.2015.09.076 26670058

[28] Eleid MF, Sorajja P, Michelena HI, Malouf JF, Scott CG, Pellikka PA. Flow-gradient patterns in severe aortic stenosis with preserved ejection fraction: clinical characteristics and predictors of survival. Circulation. Lippincott Williams & Wilkins; 2013;128:1781–9. 10.1161/CIRCULATIONAHA.113.003695 PMC3929123 24048203

[29] Clavel M-A, Dumesnil JG, Capoulade R, Mathieu P, Sénéchal M, Pibarot P (2012). Outcome of patients with aortic stenosis, small valve area, and low-flow, low-gradient despite preserved left ventricular ejection fraction. J Am Coll Cardiol.

[30] O’Sullivan CJ, Stortecky S, Heg D, Pilgrim T, Hosek N, Buellesfeld L, et al. Clinical outcomes of patients with low-flow, low-gradient, severe aortic stenosis and either preserved or reduced ejection fraction undergoing transcatheter aortic valve implantation. European Heart Journal. The Oxford University Press; 2013;34:3437–50. 10.1093/eurheartj/eht408 24096324

[31] Ozkan A, Hachamovitch R, Kapadia SR, Tuzcu EM, Marwick TH. Impact of aortic valve replacement on outcome of symptomatic patients with severe aortic stenosis with low gradient and preserved left ventricular ejection fraction. Circulation. Lippincott Williams & Wilkins; 2013;128:622–31. 10.1161/CIRCULATIONAHA.112.001094 23812184

[32] Mehrotra P, Jansen K, Flynn AW, Tan TC, Elmariah S, Picard MH, et al. Differential left ventricular remodelling and longitudinal function distinguishes low flow from normal-flow preserved ejection fraction low-gradient severe aortic stenosis. European Heart Journal. The Oxford University Press; 2013;34:1906–14. 10.1093/eurheartj/eht094 PMC3858103 23533186

[33] Jander N, Hochholzer W, Kaufmann BA, Bahlmann E, Gerdts E, Boman K, et al. Velocity ratio predicts outcomes in patients with low gradient severe aortic stenosis and preserved EF. Br Heart J. BMJ Publishing Group Ltd and British Cardiovascular Society; 2014;100:1946–53. 10.1136/heartjnl-2014-305763 25217488

[34] Lancellotti P, Lebois F, Simon M, Tombeux C, Chauvel C, Pierard LA (2005). Prognostic importance of quantitative exercise Doppler echocardiography in asymptomatic valvular aortic stenosis. Circulation.

[35] Maréchaux S, Hachicha Z, Bellouin A, Dumesnil JG, Meimoun P, Pasquet A, et al. Usefulness of exercise-stress echocardiography for risk stratification of true asymptomatic patients with aortic valve stenosis. European Heart Journal. The Oxford University Press; 2010;31:1390–7. 10.1093/eurheartj/ehq076 PMC2878968 20308041

[36] Saura D, de la Morena G, Flores-Blanco PJ, Oliva MJ, Caballero L, González-Carrillo J (2015). Aortic valve stenosis planimetry by means of three-dimensional transesophageal echocardiography in the real clinical setting: feasibility, reliability and systematic deviations. Echocardiography.

[37] Qizilbash B, Couture P, Denault A. Impact of perioperative transesophageal echocardiography in aortic valve replacement. Semin Cardiothorac Vasc Anesth. SAGE Publications; 2007;11:288–300. 10.1177/1089253207311789 18270194

[38] Michelena HI, Abel MD, Suri RM, Freeman WK, Click RL, Sundt TM (2010). Intraoperative echocardiography in valvular heart disease: an evidence-based appraisal. Mayo Clin Proc.

[39] Ionescu AA, West RR, Proudman C, Butchart EG, Fraser AG (2001). Prospective study of routine perioperative transesophageal echocardiography for elective valve replacement: clinical impact and cost-saving implications. J Am Soc Echocardiogr.

[40] Flachskampf FA, Wouters PF, Edvardsen T, Evangelista A, Habib G, Hoffman P (2014). Recommendations for transoesophageal echocardiography: EACVI update 2014. Eur Heart J - Cardiovasc Imaging.

[41] Hahn RT, Little SH, Monaghan MJ, Kodali SK, Williams M, Leon MB, et al. Recommendations for comprehensive intraprocedural echocardiographic imaging during TAVR. JACC Cardiovasc Imaging. 2015. pp. 261–87. 10.1016/j.jcmg.2014.12.014 25772834

[42] Wang H, Hanna JM, Ganapathi A, Keenan JE, Hurwitz LM, Vavalle JP (2015). Comparison of aortic annulus size by transesophageal echocardiography and computed tomography angiography with direct surgical measurement. Am J Cardiol.

[43] Tsuneyoshi H, Komiya T, Shimamoto T (2016). Accuracy of aortic annulus diameter measurement: comparison of multi-detector CT, Two- and three-dimensional echocardiography. J Card Surg.

[44] Altiok E, Koos R, Schröder J, Brehmer K, Hamada S, Becker M, et al. Comparison of two-dimensional and three-dimensional imaging techniques for measurement of aortic annulus diameters before transcatheter aortic valve implantation. Heart. BMJ Publishing Group Ltd and British Cardiovascular Society; 2011;97:1578–84. 10.1136/hrt.2011.223974 21700756

[45.••] Rosenhek R, Binder T, Porenta G, Lang I, Christ G, Schemper M, et al. Predictors of outcome in severe, asymptomatic aortic stenosis. N Engl J Med. 2000;343:611–7. **This seminal paper suggested the strong prognostic importance of aortic valve calcification, outperforming traditional assessment of hemodynamic severity**. 10.1056/NEJM200008313430903 10965007

[46.••] Rosenhek R, Klaar U, Schemper M, Scholten C, Heger M, Gabriel H, et al. Mild and moderate aortic stenosis. Natural history and risk stratification by echocardiography. Eur Heart J. The Oxford University Press; 2004;25:199–205. **The value of using the above echocardiography-based calcium scoring system was subsequently demonstrated in patients with mild and moderate AS**. 10.1016/j.ehj.2003.12.002 14972419

[47] Messika-Zeitoun D, Aubry M-C, Detaint D, Bielak LF, Peyser PA, Sheedy PF, et al. Evaluation and clinical implications of aortic valve calcification measured by electron-beam computed tomography. Circulation. Lippincott Williams & Wilkins; 2004;110:356–62. 10.1161/01.CIR.0000135469.82545.D0 15249504

[48] Cowell SJ, Newby DE, Burton J, White A, Northridge DB, Boon NA (2003). Aortic valve calcification on computed tomography predicts the severity of aortic stenosis. Clin Radiol.

[49] Cueff C, Serfaty J-M, Cimadevilla C, Laissy J-P, Himbert D, Tubach F, et al. Measurement of aortic valve calcification using multislice computed tomography: correlation with haemodynamic severity of aortic stenosis and clinical implication for patients with low ejection fraction. Heart. BMJ Publishing Group Ltd and British Cardiovascular Society; 2011;97:721–6. 10.1136/hrt.2010.198853 20720250

[50] Messika-Zeitoun D, Bielak LF, Peyser PA, Sheedy PF, Turner ST, Nkomo VT, et al. Aortic valve calcification: determinants and progression in the population. Arterioscler. Thromb. Vasc. Biol. Lippincott Williams & Wilkins; 2007;27:642–8. 10.1161/01.ATV.0000255952.47980.c2 17185617

[51] Nguyen V, Cimadevilla C, Estellat C, Codogno I, Huart V, Benessiano J, et al. Haemodynamic and anatomic progression of aortic stenosis. Br Heart J. BMJ Publishing Group Ltd and British Cardiovascular Society; 2015;101:943–7. 10.1136/heartjnl-2014-307154 25655063

[52] Dweck MR, Jenkins WSA, Vesey AT, Pringle MAH, Chin CWL, Malley TS, et al. 18F-sodium fluoride uptake is a marker of active calcification and disease progression in patients with aortic stenosis. Circ Cardiovasc Imaging. Lippincott Williams & Wilkins; 2014;7:371–8. 10.1161/CIRCIMAGING.113.001508 24508669

[53.••] Clavel M-A, Pibarot P, Messika-Zeitoun D, Capoulade R, Malouf J, Aggarval S, et al. Impact of aortic valve calcification, as measured by MDCT, on survival in patients with aortic stenosis: results of an international registry study. J Am Coll Cardiol. 2014;64:1202–13. **This multicentre observational study showed that previously defined sex-specific values for severe aortic stenosis based on CT assessment of aortic valve calcification provided incremental prognostic information beyond echo-derived measures**. 10.1016/j.jacc.2014.05.066 PMC4391203 25236511

[54] Aksoy O, Cam A, Agarwal S, Ige M, Yousefzai R, Singh D, et al. Significance of aortic valve calcification in patients with low-gradient low-flow aortic stenosis. Clin Cardiol. Wiley Periodicals, Inc; 2014;37:26–31. 10.1002/clc.22212 PMC6649593 24122890

[55] Clavel M-A, Malouf J, Messika-Zeitoun D, Araoz PA, Michelena HI, Enriquez-Sarano M (2015). Aortic valve area calculation in aortic stenosis by CT and Doppler echocardiography. JACC Cardiovasc Imaging.

[56] Chin CWL, Khaw HJ, Luo E, Tan S, White AC, Newby DE (2014). Echocardiography underestimates stroke volume and aortic valve area: implications for patients with small-area low-gradient aortic stenosis. Can J Cardiol.

[57] Irkle A, Vesey AT, Lewis DY, Skepper JN, Bird JLE, Dweck MR, et al. Identifying active vascular microcalcification by (18)F-sodium fluoride positron emission tomography. Nat Commun. Nature Publishing Group; 2015;6:7495. 10.1038/ncomms8495 PMC4506997 26151378

[58] Dweck MR, Jones C, Joshi NV, Fletcher AM, Richardson H, White A, et al. Assessment of valvular calcification and inflammation by positron emission tomography in patients with aortic stenosis. Circulation. Lippincott Williams & Wilkins; 2012;125:76–86. 10.1161/CIRCULATIONAHA.111.051052 22090163

[59] Jenkins WSA, Vesey AT, Shah ASV, Pawade TA, Chin CWL, White AC (2015). Valvular (18)F-fluoride and (18)F-fluorodeoxyglucose uptake predict disease progression and clinical outcome in patients with aortic stenosis. J Am Coll Cardiol.

[60] Dweck MR, Khaw HJ, Sng GKZ, Luo ELC, Baird A, Williams MC, et al. Aortic stenosis, atherosclerosis, and skeletal bone: is there a common link with calcification and inflammation? European Heart Journal. The Oxford University Press; 2013;34:1567–74. 10.1093/eurheartj/eht034 23391586

[61] Tawakol A, Migrino RQ, Bashian GG, Bedri S, Vermylen D, Cury RC (2006). In vivo 18F-fluorodeoxyglucose positron emission tomography imaging provides a noninvasive measure of carotid plaque inflammation in patients. J Am Coll Cardiol.

[62] Gunther S, Grossman W (1979). Determinants of ventricular function in pressure-overload hypertrophy in man. Circulation.

[63] Salcedo EE, Korzick DH, Currie PJ, Stewart WJ, Lever HM, Goormastic M (1989). Determinants of left ventricular hypertrophy in patients with aortic stenosis. Cleve Clin J Med.

[64] Hein S, Arnon E, Kostin S, Schönburg M, Elsässer A, Polyakova V (2003). Progression from compensated hypertrophy to failure in the pressure-overloaded human heart: structural deterioration and compensatory mechanisms. Circulation.

[65] Yilmaz A, Kindermann I, Kindermann M, Mahfoud F, Ukena C, Athanasiadis A, et al. Comparative evaluation of left and right ventricular endomyocardial biopsy: differences in complication rate and diagnostic performance. Circulation. Lippincott Williams & Wilkins; 2010;122:900–9. 10.1161/CIRCULATIONAHA.109.924167 20713901

[66] Kim RJ, Fieno DS, Parrish TB, Harris K, Chen EL, Simonetti O (1999). Relationship of MRI delayed contrast enhancement to irreversible injury, infarct age, and contractile function. Circulation.

[67] de Jong S, van Veen TAB, de Bakker JMT, Vos MA, van Rijen HVM (2011). Biomarkers of myocardial fibrosis. J Cardiovasc Pharmacol.

[68] Wu E, Judd RM, Vargas JD, Klocke FJ, Bonow RO, Kim RJ. Visualisation of presence, location, and transmural extent of healed Q-wave and non-Q-wave myocardial infarction. Lancet. Elsevier; 2001;357:21–8. 10.1016/S0140-6736(00)03567-4 11197356

[69] Rudolph A, Abdel-Aty H, Bohl S, Boyé P, Zagrosek A, Dietz R (2009). Noninvasive detection of fibrosis applying contrast-enhanced cardiac magnetic resonance in different forms of left ventricular hypertrophy relation to remodeling. J Am Coll Cardiol.

[70.•] Dweck MR, Joshi S, Murigu T, Alpendurada F, Jabbour A, Melina G, et al. Midwall fibrosis is an independent predictor of mortality in patients with aortic stenosis. J Am Coll Cardiol. 2011;58:1271–9. **The presence of mid-wall fibrosis on CMR was associated with an eight-fold increase in all-cause mortality**. 10.1016/j.jacc.2011.03.064 21903062

[71.•] Barone-Rochette G, Piérard S, de de Meester Ravenstein C, Seldrum S, Melchior J, Maes F, et al. Prognostic significance of LGE by CMR in aortic stenosis patients undergoing valve replacement. J Am Coll Cardiol. 2014;64:144–54. **Again, mid-wall fibrosis is an independent predictor of all-cause mortality following AVR**. 10.1016/j.jacc.2014.02.612 25011718

[72] . Weidemann F, Herrmann S, Störk S, Niemann M, Frantz S, Lange V, et al. Impact of myocardial fibrosis in patients with symptomatic severe aortic stenosis. Circulation. Lippincott Williams & Wilkins; 2009;120:577–84. 10.1161/CIRCULATIONAHA.108.847772 19652094

[73.•] Azevedo CF, Nigri M, Higuchi ML, Pomerantzeff PM, Spina GS, Sampaio RO, et al. Prognostic significance of myocardial fibrosis quantification by histopathology and magnetic resonance imaging in patients with severe aortic valve disease. JACC. 2010;56:278–87. **A key study showing that mid-wall fibrosis is associated with worse improvement in LV function post-AVR and is an independent predictor of long-term survival**. 10.1016/j.jacc.2009.12.074 20633819

[74] Chin CWL, Shah ASV, McAllister DA, Joanna Cowell S, Alam S, Langrish JP (2014). High-sensitivity troponin I concentrations are a marker of an advanced hypertrophic response and adverse outcomes in patients with aortic stenosis. Eur Heart J.

[75] Krayenbeuhl HP, Hess OM, Monrad ES, Schneider J, Mall G, Turina M (1989). Left-ventricular myocardial structure in aortic-valve disease before, intermediate, and late after aortic-valve replacement. Circulation.

[76] Higgins DM, Moon JC. Review of T1 mapping methods: comparative effectiveness including reproducibility issues. Curr Cardiovasc Imaging Rep. Springer US; 2014;7:1–10.

[77] Khawaja AZ, Cassidy DB, Shakarchi Al J, McGrogan DG, Inston NG, Jones RG. Revisiting the risks of MRI with gadolinium based contrast agents-review of literature and guidelines. Insights Imaging. Springer Berlin Heidelberg; 2015;6:553–8. 10.1007/s13244-015-0420-2 PMC4569598 26253982

[78.•] Chin CWL, Semple S, Malley T, White AC, Mirsadraee S, Weale PJ, et al. Optimization and comparison of myocardial T1 techniques at 3T in patients with aortic stenosis. Eur Heart J- Cardiovasc Imaging. 2014;15:556–65. **This paper demonstrated superior intra, inter observer and scan-rescan reproducibility in ECV assessment of patients with aortic stenosis**. 10.1093/ehjci/jet245 PMC3979453 24282220

[79] Flett AS, Hayward MP, Ashworth MT, Hansen MS, Taylor AM, Elliott PM, et al. Equilibrium contrast cardiovascular magnetic resonance for the measurement of diffuse myocardial fibrosis: preliminary validation in humans. Circulation. Lippincott Williams & Wilkins; 2010;122:138–44. 10.1161/CIRCULATIONAHA.109.930636 20585010

[80] Fontana M, White SK, Banypersad SM. Comparison of T1 mapping techniques for ECV quantification. Histological validation and reproducibility of ShMOLLI versus multibreath-hold T1 quantification …. J Cardiovasc Magn …. 2012. 10.1186/1532-429X-14-88 PMC3552758 23272651

[81] White SK, Sado DM, Fontana M, Banypersad SM, Maestrini V, Flett AS (2013). T1 mapping for myocardial extracellular volume measurement by CMR: bolus only versus primed infusion technique. JACC Cardiovasc Imaging.

[82] Kammerlander AA, Marzluf BA, Zotter-Tufaro C, Aschauer S, Duca F, Bachmann A (2016). T1 mapping by CMR imaging: from histological validation to clinical implication. JACC Cardiovasc Imaging.

[83] Bull S, White SK, Piechnik SK, Flett AS, Ferreira VM, Loudon M, et al. Human non-contrast T1 values and correlation with histology in diffuse fibrosis. Heart. BMJ Publishing Group Ltd and British Cardiovascular Society; 2013;99:932–7. 10.1136/heartjnl-2012-303052 PMC3686317 23349348

[84] Lee S-P, Lee W, Lee JM, Park E-A, Kim H-K, Kim Y-J, et al. Assessment of diffuse myocardial fibrosis by using MR imaging in asymptomatic patients with aortic stenosis. Radiology. Radiological Society of North America; 2015;274:359–69. 10.1148/radiol.14141120 25251584

[85] Puntmann VO, Carr-White G, Jabbour A, Yu C-Y, Gebker R, Kelle S (2016). T1-mapping and outcome in nonischemic cardiomyopathy: all-cause mortality and heart failure. JACC Cardiovasc Imaging.

[86] Flett AS, Sado DM, Quarta G, Mirabel M, Pellerin D, Herrey AS, et al. Diffuse myocardial fibrosis in severe aortic stenosis: an equilibrium contrast cardiovascular magnetic resonance study. European Heart Journal - Cardiovascular Imaging. The Oxford University Press; 2012;13:jes102–826. 10.1093/ehjci/jes102 22634740

[87] Hinojar R, Varma N, Child N, Goodman B, Jabbour A, Yu C-Y, et al. T1 Mapping in discrimination of hypertrophic phenotypes: hypertensive heart disease and hypertrophic cardiomyopathy: findings from the International T1 Multicenter Cardiovascular Magnetic Resonance Study. Circ Cardiovasc Imaging. Lippincott Williams & Wilkins; 2015;8:e003285. 10.1161/CIRCIMAGING.115.003285 26659373

[88] Chin CWL, Messika-Zeitoun D, Shah ASV, Lefevre G, Bailleul S, Yeung ENW, et al. A clinical risk score of myocardial fibrosis predicts adverse outcomes in aortic stenosis. European Heart Journal. The Oxford University Press; 2015;:ehv525. 10.1093/eurheartj/ehv525 PMC4761400 26491110

[89] Le Ven F, Tizón-Marcos H, Fuchs C, Mathieu P, Pibarot P, Larose E (2014). Valve tissue characterization by magnetic resonance imaging in calcific aortic valve disease. Can J Cardiol.

[90] Lancellotti P, Tribouilloy C, Hagendorff A, Popescu BA, Edvardsen T, Pierard LA, et al. Recommendations for the echocardiographic assessment of native valvular regurgitation: an executive summary from the European Association of Cardiovascular Imaging. European Heart Journal - Cardiovascular Imaging. Oxford University Press; 2013;14:611–44. 10.1093/ehjci/jet105 23733442

[91] Perry GJ, Helmcke F, Nanda NC, Byard C, Soto B (1987). Evaluation of aortic insufficiency by Doppler color flow mapping. JACC.

[92] Fang L, Hsiung MC, Miller AP, Nanda NC, Yin WH, Young MS, et al. Assessment of aortic regurgitation by live three-dimensional transthoracic echocardiographic measurements of vena contracta area: usefulness and validation. Echocardiography. Blackwell Science Inc; 2005;22:775–81. 10.1111/j.1540-8175.2005.00171.x 16194172

[93] Tribouilloy C, Avinée P, Shen WF, Rey JL, Slama M, Lesbre JP. End diastolic flow velocity just beneath the aortic isthmus assessed by pulsed Doppler echocardiography: a new predictor of the aortic regurgitant fraction. Br Heart J. BMJ Group; 1991;65:37–40. 10.1136/hrt.65.1.37 PMC1024460 1993128

[94] Choi J, Hong G-R, Kim M, Cho IJ, Shim CY, Chang H-J, et al. Automatic quantification of aortic regurgitation using 3D full volume color doppler echocardiography: a validation study with cardiac magnetic resonance imaging. Int J Cardiovasc Imaging. Springer Netherlands; 2015;31:1379–89. 10.1007/s10554-015-0707-x 26164059

[95] Tornos MP, Olona M, Permanyer-Miralda G, Herrejon MP, Camprecios M, Evangelista A (1995). Clinical outcome of severe asymptomatic chronic aortic regurgitation: a long-term prospective follow-up study. Am Heart J.

[96] Dujardin KS, Enriquez-Sarano M, Schaff HV, Bailey KR, Seward JB, Tajik AJ (1999). Mortality and morbidity of aortic regurgitation in clinical practice. A long-term follow-up study. Circulation.

[97] Bonow RO, Lakatos E, Maron BJ, Epstein SE (1991). Serial long-term assessment of the natural history of asymptomatic patients with chronic aortic regurgitation and normal left ventricular systolic function. Circulation.

[98] Turina J, Milincic J, Seifert B, Turina M. Valve replacement in chronic aortic regurgitation. True predictors of survival after extended follow-up. Circulation. 1998;98:II100–6–discussionII106–7. 9852889

[99] Chaliki HP, Mohty D, Avierinos J-F, Scott CG, Schaff HV, Tajik AJ (2002). Outcomes after aortic valve replacement in patients with severe aortic regurgitation and markedly reduced left ventricular function. Circulation.

[100] Marciniak A, Sutherland GR, Marciniak M, Claus P, Bijnens B, Jahangiri M. Myocardial deformation abnormalities in patients with aortic regurgitation: a strain rate imaging study. Eur J Echocardiogr. The Oxford University Press; 2009;10:112–9. 10.1093/ejechocard/jen185 18579501

[101] Smedsrud MK, Pettersen E, Gjesdal O, Svennevig JL, Andersen K, Ihlen H (2011). Detection of left ventricular dysfunction by global longitudinal systolic strain in patients with chronic aortic regurgitation. J Am Soc Echocardiogr.

[102] Ewe SH, Haeck MLA, Ng ACT, Witkowski TG, Auger D, Leong DP, et al. Detection of subtle left ventricular systolic dysfunction in patients with significant aortic regurgitation and preserved left ventricular ejection fraction: speckle tracking echocardiographic analysis. European Heart Journal - Cardiovascular Imaging. Oxford University Press; 2015;16:992–9. 10.1093/ehjci/jev019 25733208

[103] Park SH, Yang YA, Kim KY, Park SM, Kim HN, Kim JH (2015). Left ventricular strain as predictor of chronic aortic regurgitation. J Cardiovasc Ultrasound.

[104] David TE. Surgical treatment of aortic valve disease. Nat Rev Cardiol. Nature Publishing Group; 2013;10:375–86. 10.1038/nrcardio.2013.72 23670613

[105] Bellenger NG, Burgess MI, Ray SG, Lahiri A, Coats AJ, Cleland JG, et al. Comparison of left ventricular ejection fraction and volumes in heart failure by echocardiography, radionuclide ventriculography and cardiovascular magnetic resonance; are they interchangeable? European Heart Journal. The Oxford University Press; 2000;21:1387–96. 10.1053/euhj.2000.2011 10952828

[106.••] Myerson SG, d’Arcy J, Mohiaddin R, Greenwood JP, Karamitsos TD, Francis JM, et al. Aortic regurgitation quantification using cardiovascular magnetic resonance: association with clinical outcome. Circulation. Lippincott Williams & Wilkins; 2012;126:1452–60. **Regurgitant fraction calculated by phase-encoded velocity mapping can classify AR severity with high accuracy and is strongly predictive of progression to symptoms or AVR within 3 years, although the RV cut-off appeared to be lower that with echocardiography**. 10.1161/CIRCULATIONAHA.111.083600 22879371

[107] Chaturvedi A, Hamilton-Craig C, Cawley PJ, Mitsumori LM, Otto CM, Maki JH. Quantitating aortic regurgitation by cardiovascular magnetic resonance: significant variations due to slice location and breath holding. Eur Radiol. Springer Berlin Heidelberg; 2015:1–10. 10.1007/s00330-015-4120-6 26634930

[108] Cawley PJ, Hamilton-Craig C, Owens DS, Krieger EV, Strugnell WE, Mitsumori L, et al. Prospective comparison of valve regurgitation quantitation by cardiac magnetic resonance imaging and transthoracic echocardiography. Circ Cardiovasc Imaging. Lippincott Williams & Wilkins; 2013;6:48–57. 10.1161/CIRCIMAGING.112.975623 23212272

[109] Honda N, Machida K, Hashimoto M, Mamiya T, Takahashi T, Kamano T (1993). Aortic regurgitation: quantitation with MR imaging velocity mapping. Radiology.

[110] Søndergaard L, Lindvig K, Hildebrandt P, Thomsen C, Ståhlberg F, Joen T (1993). Quantification of aortic regurgitation by magnetic resonance velocity mapping. Am Heart J.

[111] Gabriel RS, Renapurkar R, Bolen MA, Verhaert D, Leiber M, Flamm SD (2011). Comparison of severity of aortic regurgitation by cardiovascular magnetic resonance versus transthoracic echocardiography. Am J Cardiol.

